# Recent aspects on epidemiology, clinical disease, and genetic diversity of *Toxoplasma gondii* infections in Australasian marsupials

**DOI:** 10.1186/s13071-021-04793-4

**Published:** 2021-06-05

**Authors:** Jitender P. Dubey, Fernando H. A. Murata, Camila K. Cerqueira-Cézar, Oliver C. H. Kwok, Chunlei Su, Michael E. Grigg

**Affiliations:** 1 United States Department of Agriculture, Agricultural Research Service, Beltsville Agricultural Research Center, Animal Parasitic Diseases Laboratory, Building 1001, Beltsville, MD 20705-2350 USA; 2 Department of Microbiology, University of Tennessee, Knoxville, TN 37996-0845 USA; 3 Molecular Parasitology Section, Laboratory of Parasitic Diseases, National Institute of Allergy and Infectious Diseases, National Institutes of Health, Bethesda, MD 20895 USA

**Keywords:** *Toxoplasma gondii*, Marsupials, Genotype, Prevalence, Clinical disease, Diagnosis

## Abstract

**Background:**

*Toxoplasma gondii* infections are common in humans and animals worldwide. Among all intermediate hosts of *T. gondii*, captive marsupials from Australia and New Zealand are highly susceptible to clinical toxoplasmosis. However, most free-range marsupials establish chronic *T. gondii* infection. Infected marsupial meat may serve as a source of *T. gondii* infection for humans. Differences in mortality patterns in different species of kangaroos and other marsupials are not fully understood. Lifestyle, habitat, and the genotype of *T. gondii* are predicted to be risk factors. For example, koalas are rarely exposed to *T. gondii* because they live on treetops whereas wallabies on land are frequently exposed to infection.

**Methods:**

The present review summarizes worldwide information on the prevalence of clinical and subclinical infections, epidemiology, and genetic diversity of *T. gondii* infecting Australasian marsupials in their native habitat and among exported animals over the past decade. The role of genetic types of *T. gondii* and clinical disease is discussed.

**Results:**

Fatal toxoplasmosis has been diagnosed in captive Australasian marsupials in Argentina, Chile, China, Germany, Hungary, Japan, Spain, Turkey, and the USA. Most deaths occurred because of disseminated toxoplasmosis. Genetic characterization of *T. gondii* strains isolated from fatal marsupial infections identified Type III as well as atypical, nonclonal genotypes. Fatal toxoplasmosis was also diagnosed in free-ranging wombats (*Vombatus ursinus*) in Australia. Genetic characterization of DNA amplified directly from host tissues of subclinical culled kangaroos at slaughter identified many mixed-strain infections with both atypical and recombinant genotypes of *T. gondii*.

**Conclusions:**

Most Australasian marsupials in their native land, Australia and New Zealand, have high prevalence of *T. gondii*, and kangaroo meat can be a source of infection for humans if consumed uncooked/undercooked. The genotypes prevalent in kangaroos in Australia and New Zealand were genetically distinct from those isolated or genotyped from most macropods in the USA and other countries. Thus, clinical toxoplasmosis in marsupials imported from Australia is most likely to occur from infections acquired after importation.

**Graphic abstract:**

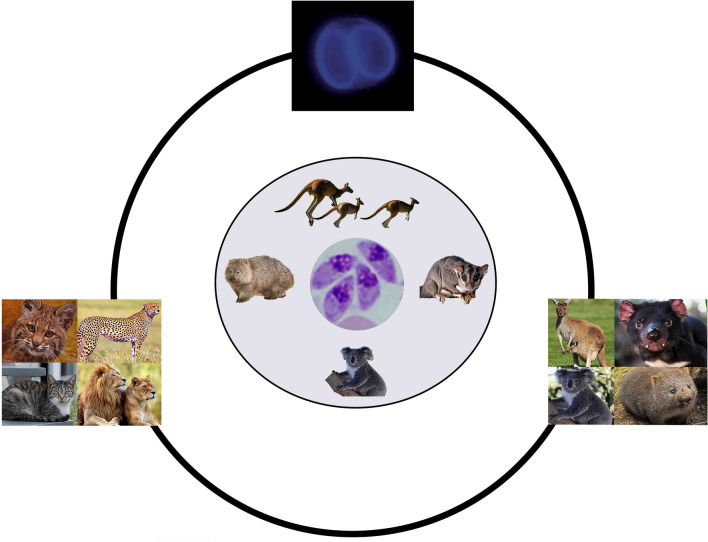

## Background

It has been known for many decades that *Toxoplasma gondii* can cause serious illness in Australasian marsupials [1]. Much information was gained when a Registry for Comparative Pathology was established at the Taranga Zoo, Sydney, Australia, through the efforts of now late Dr. Bill Hartley [2]. A comprehensive report histologically diagnosed toxoplasmosis in 79 marsupials [3]. Since then, there have been numerous reports of deaths in captive Australasian marsupials in zoos worldwide [2, 4], suggesting that these animals are highly susceptible to clinical toxoplasmosis. However, serological surveys of free-ranging populations of marsupial species suggest that prevalence of infection can be high and that death from toxoplasmosis in the wild may be more dependent on other factors, including host species and the strain of *T. gondii*. Because infected kangaroo meat may serve as a source of infection for humans, understanding the pathogenesis and prevalence of toxoplasmosis in marsupials is of considerable interest. This report is divided into two sections, captive and free-range (FR) animals.

Initially, marsupials in zoos around the world were imported from Australia and New Zealand. Because these animals are stressed easily, many of them died in zoos because of the stress of transportation and relocation. Some zoos now have their own breeding programs; however, captive marsupials continue to die of toxoplasmosis [2, 4]. There is a marked contrast regarding clinical toxoplasmosis in wallabies in the wild (FR) versus captivity; the etiology is poorly understood. The genotype of *T. gondii* found in FR versus captive marsupials could be a factor. Here, we review progress concerning genetic diversity of *T. gondii* in Australasian marsupials and the outcome of *T. gondii* infections in these animals.

### Zoo animals outside of Australia and New Zealand

Reports of clinical toxoplasmosis in the past decade are summarized in Table 1.

**Table 1:** Clinical toxoplasmosis in Australasian marsupials

Host	Country	Main findings	References
Agile wallaby (*Macropus agilis*)	USA	See text. Case 2, DNA extracted from heart tissue, genotype #263	[5]
Bennett’s wallaby (*Macropus rufogriseus*)	Germany	An 8.5-year-old male wallaby had fever, neurological and ophthalmic signs. Serological testing by IHA^a^ revealed a titer of > 1:1024. The animal recovered after subcutaneous injections of trimethoprim/sulfadoxin (15 mg/kg) for 7 days	[6]
Bennett’s wallaby (*Macropus rufogriseus*)	Spain	9-year-old male. Histologically confirmed disseminated toxoplasmosis, including lymph nodes. *T. gondii* DNA extracted from the brain was PCR positive and found to be atypical genotype based on 15 microsatellite markers	[7]
Bennett’s wallaby (*Macropus rufogriseus*)	USA	9 of the 10 wallabies imported from New Zealand to a zoo in Virginia died of acute toxoplasmosis; all were seronegative at the time of import. Myositis, encephalitis, myocarditis, and interstitial pneumonia were the main lesions and the diagnosis was confirmed by IHC and PCR. Genotyping of the isolates suggested 2 sources of infection acquired in the USA. From 6 wallaby tissues, the DNA was typed as PCR-RFLP-ToxoDB genotype #263, and from the 3 others the genotype was ToxoDB #4, commonly found in wildlife	[8]
Bennett’s wallaby (*Macropus rufogriseus*)	USA	See text. Case 3, DNA extracted from heart and lung tissues, genotype #2	[5]
Eastern grey kangaroo (*Macropus giganteus*)	Argentina	Non-suppurative encephalitis was found at necropsy of 1 captive kangaroo that died. Diagnosis was confirmed by IHC and PCR. *T. gondii* was isolated from brain and diaphragm of the kangaroo. Genotyping using 9 PCR-RFLP markers from heart and hind limb revealed genotype II	[9]
Eastern grey kangaroo (*Macropus giganteus*)	China	6-year-old female born in captivity in China died suddenly, probably of bacterial infection. Tissue cysts were found in tongue and diaphragm. The animal was MAT-positive and *T. gondii* DNA was detected in tissue extract. Viable *T. gondii* (TgRooCHn1) was isolated by bioassay in mice. PCR-RFLP-ToxoDB genotype was #292	[10]
Eastern grey kangaroo (*Macropus giganteus*)	Japan	Carcass of 1 adult, male kangaroo with neurological signs, anorexia, diarrhea, arrhythmia, was necropsied. Lesions were seen in lungs, brain and heart. Diagnosis confirmed by IHC	[11]
Red kangaroo (*Macropus rufus*)	Argentina	Non-suppurative encephalitis was found at necropsy of 1 captive kangaroo that died in captivity. Focal necrosis in heart and striated muscles with presence of *T. gondii*-like tachyzoites and cysts. Diagnosis was confirmed by IHC and PCR. *T. gondii* was isolated from brain and diaphragm of the kangaroo. Genotyping using 9 PCR-RFLP markers from heart and hind limb revealed genotype III	[9]
Red kangaroo (*Macropus rufus*)	Chile	1 adult female in zoo was found dead. Toxoplasmosis associated lesions were seen in heart, and lung. The diagnosis was confirmed by IHC	[12]
Red kangaroo (*Macropus rufus*)	China	5-year-old male born in captivity in China. Main lesions were pneumonitis and glomerulonephritis. Tissue cysts were found in histological sections. The animal was MAT-negative but PCR positive. Attempts to isolate *T. gondii* by bioassay in mice were unsuccessful	[10]
Red kangaroo (*Macropus rufus*)	USA	1-year-old male with a 1-day history of depression, labored respiration and lethargy. Disseminated toxoplasmosis including fore stomach. Gastric ulceration with concurrent amoebic infection. Diagnosis confirmed by IHC	[13]
Red kangaroo (*Macropus rufus*)	USA	See text. 8 cases 1, 4, 5, 6, 8, 9, 10, 11. Three isolates obtained (cases 1, 6, 8), the others DNA extracted from heart tissues. Genotype by 10 PCR-RFLP markers, identified three genotypes: #2 in 4 kangaroos (cases 4, 5, 6, 11), #216 in 3 kangaroos (cases 8, 9, 10), #263 in 1 kangaroo (case 1)	[5]
Tammar wallaby (*Macropus eugenii*)	Hungary	10 wallabies died of toxoplasmosis like illness; diagnosis was confirmed by IHC in 6 and histologically in 4 others	[14]
Tammar wallaby (*Macropus eugenii*)	USA	See text. Case 7, DNA extracted from heart tissue, genotype #216	[5]
Western brush wallaby (*Macropus irm*a)	Turkey	Dead adult kangaroo was necropsied. Disseminated toxoplasmosis was diagnosed histologically and confirmed by IHC and PCR. Lesions included pancreatitis, gastritis, enteritis, and encephalitis	[15]

*IHA* indirect hemagglutination test, *IHC* immunohistochemical test with *T. gondii* antibodies, *MAT* modified agglutination test, *PCR* polymerase chain reaction, *RFLP* restriction fragment length polymorphism

^a^Celloggnost-Toxoplasmosis H, Dade Behring

In addition to the data in Table 1, three separate outbreaks of systematic toxoplasmosis were diagnosed in a total of 20 marsupials that died between 2014 and 2018 in a zoo in Florida, USA [5]. The diagnosis was confirmed by IHC in 11: 1 agile wallaby (*Macropus agilis*), 8 red kangaroos (*Macropus rufus*), 1 Bennett’s wallaby (*Macropus rufogriseus*), and 1 tammar wallaby (*Macropus eugenii*). Diagnosis was confirmed by PCR and by isolation of viable *T. gondii* (Tables 1, 2, 3).

**Table 2:** Isolation and genetic characterization^a^ of viable *T. gondii* from tissues of marsupials with clinical toxoplasmosis

Host	Country	Location	Tissues^b^	Isolate designation	TOXODB genotype	References
Bennett’s wallaby (*Macropus rufogriseus*)	Argentina	La Plata	B, D	TgWb1Arg	#14	[16]
Bennett’s wallaby (*Macropus rufogriseus*)	USA	Pennsylvania	B	TgWyUs2	#2 (Type III)	[17, 18]
Bennett’s wallaby (*Macropus rufogriseus*)	USA	Pennsylvania	B	TgWyUs3	#2 (Type III)	[17, 18]
Eastern grey kangaroo (*Macropus giganteus*)	Argentina	La Plata	B, D	TgKg2Arg	Likely #285	[9, 16]
Eastern grey kangaroo (*Macropus giganteus*)	China	Henan	D, H, Sk, T	TgRooCHn1	#292	[10]
Red kangaroo (*Macropus rufus*)	Argentina	La Plata	B, D	TgKg1Arg	#2 (Type III)	[9, 16]
Red kangaroo (*Macropus rufus*)	USA	Florida	H, Lu	TgKgrFL1 from case 1	#263	[5]
Red kangaroo (*Macropus rufus*)	USA	Florida	H, Lu	TgKgrFL2 from case 6	#2 (Type III)	[5]
Red kangaroo (*Macropus rufus*)	USA	Florida	H, Lu	TgKgrFL3 from case 8	#216	[5]
Tammar wallaby (*Macropus eugenii*)	USA	Pennsylvania	B, H, Lu, Sk	TgWyUs1	#2 (Type III)	[17, 18]
Tammar wallaby (*Macropus eugenii*)	USA	Washington, DC	B, H, Li, Ln, Lu pooled	TgWyUs4	#186	[18]

^a^Ten PCR-RFLP markers (restriction fragment length polymorphism)

^b^*B* brain, *D* diaphragm, *H* heart, *Li* liver, *Ln* lymph node, *Lu* lung, *Sk* skeletal muscle, *T* tongue

**Table 3:** Genetic characterization of *T. gondii* from DNA isolated from tissues of marsupials

Host	No	Country	Location	Tissues	Method	Genotype ToxoDB or sequencing	References
Agile wallaby (*Macropus agilis*)	1	USA	Florida	H, Lu	10 PCR-RFLP markers	#263	[5]
Bennett’s wallaby (*Macropus rufogriseus*)	1	Spain	Northeast	B	15 Microsatellites markers	Atypical	[7]
Bennett’s wallaby (*Macropus rufogriseus*)	1	USA	Florida	H, Lu	10 PCR-RFLP markers	#2	[5]
Bennett’s wallaby (*Macropus rufogriseus*)	9	USA	Virginia	Li, Lu, S	10 PCR-RFLP markers	#263 in 6, #4 in 3	[8]
Common wallaroo (*Macropus robustus*)	5	Australia	WA	H, Lu, Li, S, D	B1, SAG2, SAG3	n/a	[31]
Red kangaroo (*Macropus rufus*)	6	Australia	WA	H, Lu, Li, S, D	B1, SAG2, SAG3	n/a	[31]
Red kangaroo (*Macropus rufus*)	8	USA	Florida	H, Lu	10 PCR-RFLP markers	#4 in 3, #216 in 2	[5]
Tammar wallaby (*Macropus eugenii*)	1	USA	Florida	H, Lu	10 PCR-RFLP markers	#216	[5]
Western grey kangaroo (*Macropus fuliginosus*)	8	Australia	WA	B, T, H	B1, SAG3, GRA6	n/a	[30]
Western grey kangaroo (*Macropus fuliginosus*)	5	Australia	WA	H, Lu, Li, S, D	B1, SAG2, SAG3	n/a	[31]

*B *brain, *D* diaphragm, *H *heart, *Li* liver, *Lu* lung, *S* spleen, *T* tongue, *RFLP* restriction fragment length polymorphism, *n/a* not applicable, *WA* Western Australia

The same scenario appears to be the case for marsupials in China [10]. The animals in the zoo in China were 4–6 years old and had been born in captivity from parents imported in 2000 [10]. The diagnosis was confirmed by isolation of viable *T. gondii*.

Serological monitoring indicates that not all captive marsupials die of toxoplasmosis. Antibodies to *T. gondii* were detected in a Bennett’s wallaby (*Macropus rufogriseus*) in Portugal (MAT, 1:25) [19] and in two out of two Western gray kangaroos (*Macropus fuliginosus*) in Brazil (MAT, 1:1024) [20]. *Toxoplasma gondii* antibodies (MAT, 1:3200) were also found in three of three Bennett’s wallabies (*Macropus rufogriseus*) in Spain, but these animals died subsequently [7].

### Infections in free-ranging marsupials in Australasia

#### Koalas (*Phascolarctos cinereus*)

The lack of finding antibodies (MAT, 1:40) in 157 (63 from mainland, 94 from island) koalas suggests that these animals are not readily exposed to *T. gondii* [21]; koalas are herbivores and live on trees and are not likely exposed to oocysts.

#### Kangaroos

Antibodies (in-house ELISA) to *T. gondii* were found in 34 (15.5%) of 219 western grey kangaroos (*Macropus fuliginosus*) from 7 locations around Perth, Australia [22]. The ELISA results agreed with results obtained from a total of 54 of the marsupials also tested using the commercial MAT; 47 sera tested negative by MAT and ELISA whereas 7 sera tested positive by both tests. Additionally, *T. gondii* PCR testing of hearts and brains of 62 kangaroos confirmed the serological results in 9 animals; all PCR-positive samples were also seropositive [22]. *Toxoplasma gondii* antibodies (IFA, 1:50) were also detected in 20 of 102 western grey kangaroos (*Macropus fulginosus ocydomus*) from Perth, Australia.

A study found no evidence of reproductive failure associated with *T. gondii* in western grey kangaroos from Perth, Australia. Antibodies to *T. gondii* were detected in 20 of 102 (19.7%) sera from kangaroos from 4 sites (2 golf courses and 2 reserves); the sera were tested by IFA at a dilution of 1:50 [23].

#### Wombats (*Vombatus ursinus*)

Fatal toxoplasmosis was diagnosed in eight FR wombats (*Vombatus ursinus*) that died in Tasmania or New South Wales, Australia, 2010–2013 [24]. An important feature of the lesions was necrosis and encephalitis, particularly of the thalamus. Other lesions were myocarditis and pneumonitis, and the diagnoses were confirmed by IHC and PCR [24]. PCR-RFLP typing revealed that two strains were Type II (ToxoDB genotypes #1 and #3).

#### Rock wallaby (*Petrogale penicillata*)

*Toxoplasma gondii* antibodies (MAT, 1:40) were detected in 3 out of 64 (4.6%) brush-tailed rock wallaby in Queensland, Australia [25].

#### Eastern quoll (*Dasyurus viverrinus*)

In a survey of 290 quolls sampled from four sites in Tasmania, Australia, seroprevalence varied from 5 to 100% and prevalence rates were directly associated with the presence of feral cats [26].

### Congenital/neonatal transmission in marsupials

The ingestion of oocysts from the environment is the most likely source of infection for wallabies. Little is known of neonatal toxoplasmosis in marsupials.

Fatal toxoplasmosis has been observed previously in two young *M. fuliginosus* captive joeys [27].

Evidence for vertical transmission of *T. gondii* in kangaroos was provided by an excellent study reported by Parameswaran [28]. Of the grey kangaroo *T. gondii* survey [22], the authors selected 62 dams that had young ones in their pouch [28]. Ten dams had high MAT titers (1:4096 in 5, 1:64,000 in 3, and 1:256,000 in 2); DNA of *T. gondii* was PCR-detected in all nine dams that were tested by PCR [22] and in hearts of two young joeys in pouch confirming congenital transmission of infection; however, *T. gondii* was not detected in histological sections of any of the joeys or dams consistent with the presence of subclinical infection [28]. *Toxoplasma gondii* DNA was also detected in a pouch animal from a brush-tailed bettong (*Bettongia penicillata* rat kangaroo, a small rat kangaroo) [28].

### Other marsupials

Antibodies (MAT, 1:40) to *T. gondii* were detected in 11% of 222 culled and 31% of 16 road- killed Tasmanian pademelon (*Thylogale billardierii*); cat density was associated with *T. gondii* infections in pademelons, and *T. gondii* antibodies were found in 7 of 8 feral cats [29]. In the same study, *T. gondii* antibodies were detected in 71% of 7 spotted-tail quoll (*Dasyurus maculatus*), 58% of 24 eastern quoll, 33% of 18 Tasmanian devils (*Sarcophilus harrisii*), 8% of 25 Bennett’s wallabies, and none of 14 brush tail possums (*Trichosurus vulpecula*) [29].

### Diagnosis

Among serological tests, the modified agglutination test (MAT) appears to be most sensitive for the detection of *T. gondii* antibodies [2]. The MAT detects only IgG antibodies because the mercaptoethanol used in the test destroys IgM and IgM-like substances that interfere with the test. Without the addition of mercaptoethanol, this test gives false reactions [2]. Ante-mortem diagnosis of toxoplasmosis in marsupials is problematic because they can die suddenly and before developing detectable antibodies. In a study performed in China, out of two marsupials that died of toxoplasmosis, one *Macropus giganteus* was seropositive whereas one *Macropus rufus* was seronegative [10].

Infected mammals generally develop IgM prior to the development of IgG. Therefore, a single high titer in MAT does not mean recent infection because the time of onset of IgG development in wallabies is largely unknown. The magnitude of the antibody titer by MAT only indicates exposure and not how recently the infection was acquired. In two naturally infected *Macropus rufogriseus* tested for MAT antibodies, the antibody titers remained high (> 25,600) for several months [17].

Histopathology, immunohistochemistry, and detection of DNA by PCR can aid diagnosis (Table 1). As stated earlier, there was excellent correlation between MAT, ELISA, and PCR [22].

### Treatment

None of the currently available medicines are very effective in treating clinical toxoplasmosis in marsupials. Atovaquone, pyrimethamine, trimethoprim-sulfadiazine, ponazuril, and clindamycin are some of the medicines that have been used to treat affected marsupials [6, 8, 17]. In Germany, a wallaby with neurological signs improved after treatment with trimethoprim/sulfadoxin for 7 days; the diagnosis was based on serological results and clinical signs [6]. However, chemotherapy with a combination of several anti-toxoplasmic medicines was not effective in nine macropods in the USA [8]. These differences might be related to the genotypes of *T. gondii* infecting marsupials in the USA [18]; no information was available for the macropod in Germany [6].

### Genetic diversity of *T. gondii* from marsupials

A review of the literature indicates that the FR marsupials in their native land are infected with *T. gondii* genotypes that are Type II-like or exist predominantly as recombinants of Type II or III (ToxoDB genotypes #1, #2, or #3) whereas those in captivity often reflect infection by endemic strains that are local to the geography of the zoo in which they reside. Therefore, these data are discussed in two sections. Different methods of genotyping used are also a reason to separate the discussion into two sections.

### Free-range marsupials

Multi-locus PCR-DNA sequencing was used. To our knowledge, genetic typing has only been carried out on DNA derived from cell-cultured tachyzoites from just two archived isolates of *T. gondii*, from a wallaby (*Macropus rufogriseus*) and a wombat (*Vombatus ursinus*) from Tasmania, Australia; these isolates were defined as variants of Type II [30]. They differed from clonal Type II strains because they possessed different, genetically drifted alleles at the B1, BAG1, and SRS2 genes. Both macropods from Tasmania were neurological and had histologically verified toxoplasmosis; no other details are available.

Limited genotyping of *T. gondii* DNA isolated from tissues of naturally infected, largely asymptomatic Australasian marsupials identified the same genetically drifted B1 alleles as well as infection by strains carrying Type I, variants of Type II, Type III, or some admixed combination of Type I, II, or III alleles [30, 31]. *Toxoplasma gondii* DNA was also detected by PCR in minced kangaroo meat destined for human consumption using ITS1 and B1 genes [30]. Of the DNA extracted from multiple tissues in 29 Australasian marsupials (*Macropus fuliginosus*), *T. gondii* DNA was detected in 11 kangaroos by PCR-DNA sequencing at 4 genes (ITS1, B1, SAG3, GRA6, and GRA 7) [30].

A follow-up PCR-DNA sequencing study to detect *T. gondii* DNA present in 16 healthy macropods that were culled near Menzies, Western Australia, established a high rate of co-infection with multiple genotypes of *T. gondii* infecting these animals [31]. DNA extracted from hearts, livers, lungs, spleens, and diaphragms of 6 *Macropus rufus*, 5 *Macropus fuliginosus*, and 5 *Macropus robustus* and genotyped at the typing markers B1, SAG2, and SAG3 established that all macropods were infected and 14 were co-infected with *T. gondii* strains that possessed either genetically drifted or archetypal Type II or III alleles [31]. It was also clear that among the genotypes identified, recombination was the most likely explanation responsible for the admixed nature of the allele inheritance patterns observed (Table 3). The highest rate of infected organs in decreasing order was heart, spleen, diaphragm, liver, and lung. Importantly, partitioning of distinct genetic types to individual organs was observed among the co-infected macropods [31]. This result is likely due to the sampling design of the study rather than something unique to macropods as strain partitioning between different organs has been observed among bobcats in rural Mississippi, USA, and is more likely a property of the parasite in areas of high endemicity and parasite genetic variation [32]. In conclusion, these results are important for the sylvatic transmission of *T. gondii*, because intermediate hosts co-infected with different genotypes of *T. gondii* will promote cross-fertilization during sexual reproduction, and recombinant lines were certainly identified in subclinical infections from the FR macropods studied.

### Captive marsupials

Based on characterization by PCR-RFLP 10 markers of DNA obtained from 11 viable *T. gondii* isolates, ToxoDB genotype #2 (Type III) was found in 5 and one each of ToxoDB genotypes #14, #186, #216, #292, #263, and #285 [5, 9, 10, 16–18] (Table 2). Thus, five Australasian marsupials died from infection with Type III strains, common in domestic animals, and six died from infection of atypical genotypes found routinely in wildlife in the USA. Additionally, PCR-RFLP analyses carried out on DNA extracted from host tissues of 18 other Australasian marsupials identified ToxoDB genotypes #2 in 4, #4 in 3, #216 in 3, #263 in 7, and 1 was atypical. Thus, in a total of 29 samples, ToxoDB genotypes #2 were identified in 9, #4 in 3, #14 in 1, #186 in 1, #216 in 4, #263 in 8, #285 in 1, #292 in 1, and an atypical type in 1. These results indicate that a genetically diverse set of *T. gondii* genotypes was identified infecting Australasian marsupials in captivity. Most of these genotypes are different from those found circulating in FR marsupials, which may suggest that *T. gondii* genotype plays a significant role in the mortality of these animals.

In conclusion, the genotypes prevalent in Australasian marsupials from Australia and New Zealand were genetically distinct from those isolated or genotyped from most macropods in the USA and other countries. Thus, clinical toxoplasmosis in macropods imported from Australia is most likely to occur from infections acquired after importation.

### Public health importance

Uncooked meat or any parts of kangaroos, including soft tissues (liver), should never be fed to cats; cats can excrete large numbers of *T. gondii* oocysts and further disseminate infection in the environment [2]. There is also an old report epidemiologically linking ingestion of kangaroo meat to clinical toxoplasmosis in humans in Australia [33]. The outbreak occurred among 60 people who attended a party in Brisbane in November 1994 [33]. Acute toxoplasmosis was diagnosed retrospectively in at least 12 persons. The outbreak was recognized because of accidental diagnosis of congenital toxoplasmosis in a child in the index case. An amniocentesis was performed for possible Rhesus incompatibility (RH factor) when the mother was in 34 weeks' gestation. One week later, she delivered a baby because of RH factor. At 3 months of age, *T. gondii* chorioretinitis was discovered. An enquiry into mother’s medical records and testing of archived sera revealed that she serologically converted during pregnancy and she recalled eating undercooked kangaroo meat at the party when she was 28 weeks' pregnant. She recalled having myalgia and lethargy 3 weeks after the party. She was also aware of a second women who saw a physician because she developed fever, nausea, myalgia, and arthralgia 9 days after the meal at the party. Subsequent enquiries revealed ten additional cases of acute toxoplasmosis. Although the evidence linking kangaroo meat to this outbreak is circumstantial, the message is clear that serious toxoplasmosis can result from eating contaminated foods. At this party a variety of foods were served. Retrospectively, a questionnaire returned by 38 of 46 individuals contacted indicated that 26 persons had eaten rare kangaroo meat; 10 of these persons developed clinical toxoplasmosis [33].

## Conclusion

Here we have discussed the prevalence, epidemiology, clinical disease, genetic diversity, and public health related to *T. gondii* infections in marsupials. Seroprevalence of *T. gondii* in free-range marsupials in Australia and New Zealand varied with the host species, with no infection detected in koalas, but high prevalence in kangaroos. Clinical toxoplasmosis was rare in free-range marsupials, but many captive Australasian marsupials have died in several counties. Genetic characterization of *T. gondii* strains isolated from fatal marsupial infections identified Type III as well as atypical, nonclonal genotypes. Genetic characterization of DNA amplified directly from host tissues of subclinical culled kangaroos at slaughter in Australia identified many mixed-strain infections with both atypical and recombinant genotypes of *T. gondii*.

## Data Availability

Not applicable.
